# Case Report: Left ventricular apical hypertrophy in a patient with Leopard syndrome mimicking a cardiac tumor: a diagnostic challenge resolved by multimodality imaging

**DOI:** 10.3389/fcvm.2024.1378078

**Published:** 2024-07-22

**Authors:** Hui Liu, Yue Zheng, Huaibi Huo, Xin Peng, Jun Yang, Chunyan Ma, Ting Liu

**Affiliations:** ^1^ Department of Radiology, The First Hospital of China Medical University, Shenyang, China; ^2^ Department of Radiology, The Third People’s Hospital of Chengdu, Chengdu, China; ^3^ Department of Cardiovascular Ultrasound, The First Hospital of China Medical University, Shenyang, China

**Keywords:** cardiac magnetic resonance, LEOPARD syndrome, multimodality imaging, left ventricular apical hypertrophy, coronary artery dilatation

## Abstract

**Background:**

LEOPARD syndrome (LS) is a rare genetic disorder presenting various clinical manifestations from childhood, complicating its diagnosis. In this study, we aim to refine the imaging presentation of LS and emphasize the importance of multimodality imaging in enhancing diagnostic accuracy and preventing serious cardiovascular events.

**Case:**

A 41-year-old woman was admitted to hospital with a suspected apical tumor detected by a transthoracic echocardiogram (TTE), which was later identified as apical myocardial hypertrophy through cardiac magnetic resonance imaging (CMR). She had abnormal electrocardiograms from the age of 2 years and freckles around the age of 4 years. In recent years, she has been experiencing exertional dyspnea. Supplemental coronary computer tomography angiography (CCTA) revealed diffuse coronary dilatation. Both multimodality imaging and clinical manifestations led to a suspicion of LS, which was confirmed by subsequent genetic testing. The patient declined further treatment. A 3-month follow-up CMR showed no significant change in the lesion.

**Conclusion:**

This report elucidates the diagnostic transition from an initial suspicion of an apical tumor by TTE to a definitive diagnosis of left ventricular apical hypertrophy by CMR in a 41-year-old woman with LS. It underscores the value of multimodality imaging (TTE, CCTA, CMR) in unraveling unusual cardiac manifestations in rare genetic disorders such as LS.

## Introduction

LEOPARD syndrome (LS) is a rare genetic disorder in which left ventricular hypertrophy (LVH) is the most common cardiac manifestation, although it may rarely manifest as apical cardiac hypertrophy (1, 2). The apical left ventricular (LV) region may be difficult to evaluate by a transthoracic echocardiogram (TTE) (3). Cardiac magnetic resonance (CMR) provides high spatial and temporal resolution in any plane, making it a more accurate tool to assess apical hypertrophy (4). Coronary computer tomography angiography (CCTA) may be helpful in assessing coronary atherosclerosis and anomalies. In this case report, the unusual presentation of apical hypertrophy and coronary artery dilatation was comprehensively diagnosed by TTE, CMR, and CCTA and supported by clinical manifestations and further genetic testing.

## Case report

**Figure 1:**
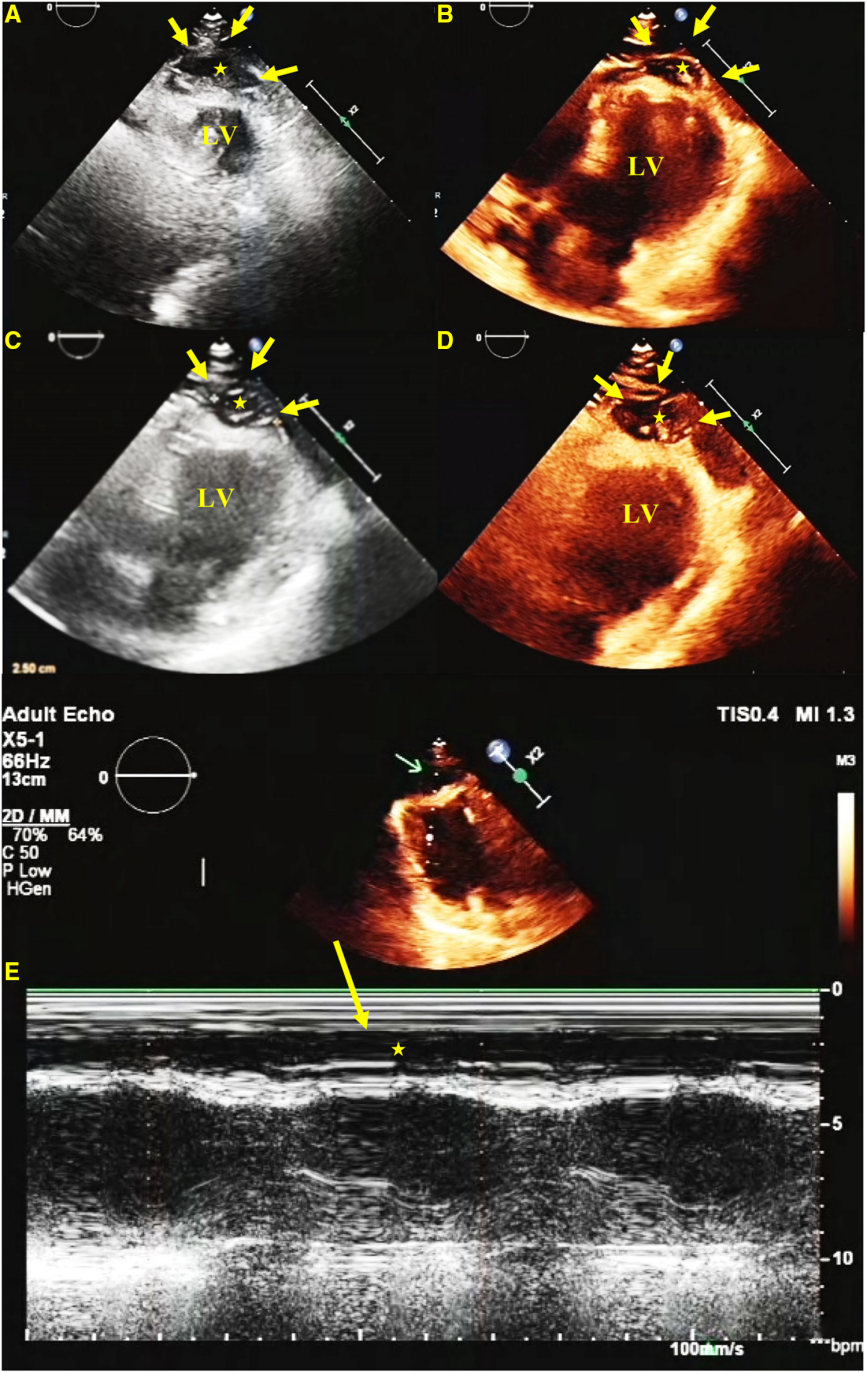
TTE. A short-axis view (**A**), a four-chamber view (**B**), and long-axis views (**C**,**D**) show a hypoechoic predominant mass in the apical region, measuring approximately 26 mm × 16 mm. The lesion protrudes toward the ventricular wall, causing compression and deformation of the myocardium. The lesion is clearly demarcated from the myocardium, and the myocardial motion is not restricted. An M-mode echocardiogram shows (**E**) abnormal apical echoes that did not change significantly during the cardiac cycle (excluding the possibility of pericardial effusion).

**Figure 2:**
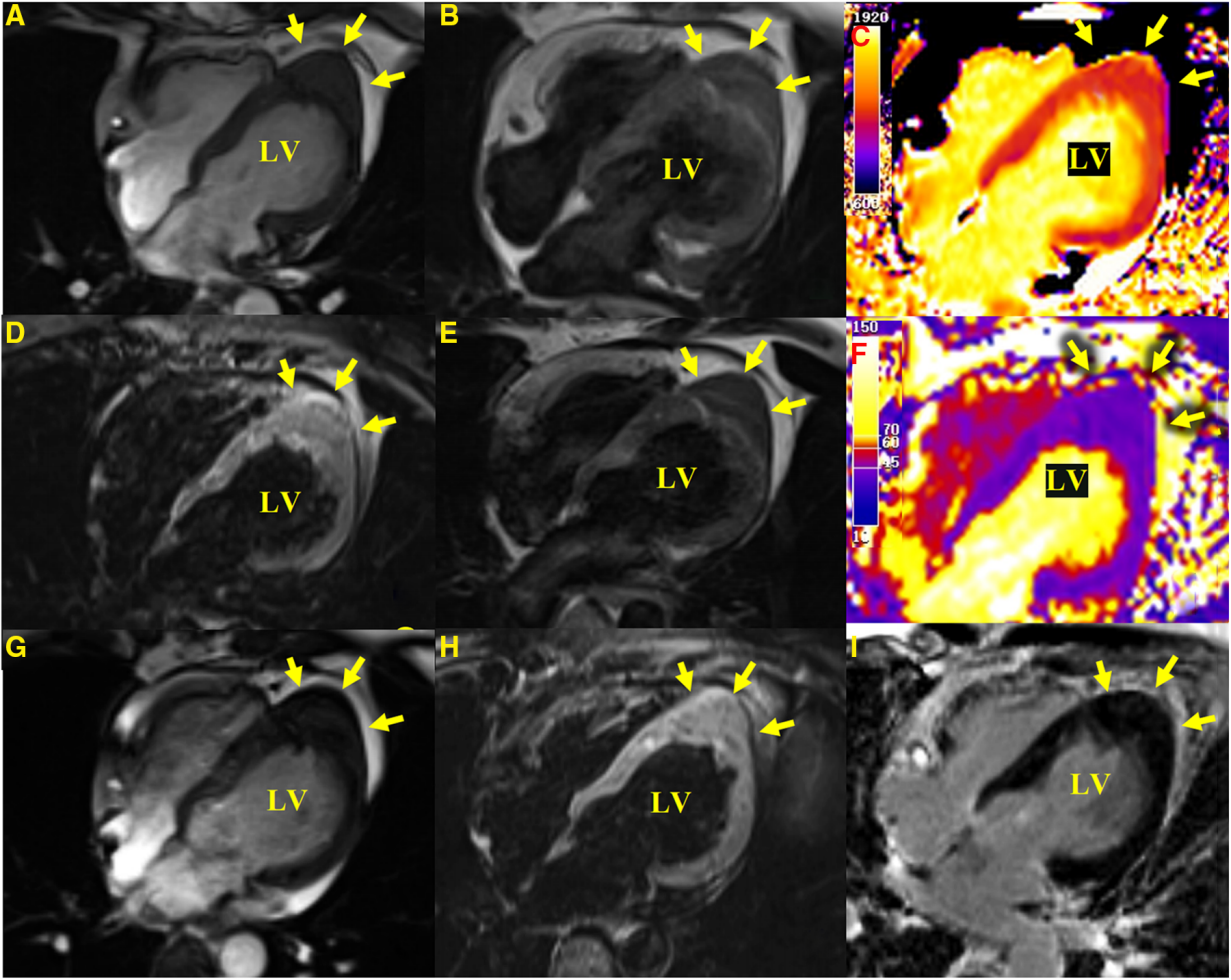
CMR images. Apical hypertrophy with a maximal thickness of 17 mm can be seen in four-chamber views in steady-state free precession cine images (**A**), a T1-weighted CMR sequence (**B**), a T2-weighted sequence (**E**), T1-mapping (**C**) (the values were approximately 1,210 ms for a normal myocardium and approximately 1,213 ms for the apical portion), a T2-mapping sequence (**F**) (the values were approximately 41 ms for a normal myocardium and approximately 40 ms for the apical portion), a fat-suppressed T2 sequence (**D**), and an LGE image (**I**) reveal similar myocardial tissue characteristics as the normal myocardium. (**G**,**H**) show a cine image and a fat-suppressed T2 sequence after 3 months with no significant change in the lesion compared with the initial presentation.

A 41-year-old woman, with a body height of 150 cm and weight of 51 kg, presented with a LV apical hypoechogenic tissue detected by TTE, initially suspected as an LV myocardial tumor (Figure 1). Subsequent CMR examination identified it as focal left ventricular hypertrophy with a mass-like protrusion of the apical myocardium measuring 17 mm in diameter, as shown in four-chamber views at the end diastole (Figure 2). Cine CMR showed that the lesion was contracting with systole and relaxing with diastole coincident with the normal myocardium, with no reverse motion observed (Supplementary Video). The valves demonstrated normal function and flow. The resting LV systolic function was normal, with an LV ejection fraction of 65%. The T1-weighted, T2-weighted, and T1/T2-mapping CMR images demonstrated myocardial characteristics similar to surrounding normal tissue (Figure 2). A linear area of hyperintensity at T1-weighted and T2-weighted CMR was seen within the lesion, becoming hypointense in fat-suppressed T2 images, suggesting myocardial fat deposition. The first-pass perfusion did not show filling defects, and late gadolinium enhancement (LGE) was unremarkable (Figure 2).

**Figure 3:**
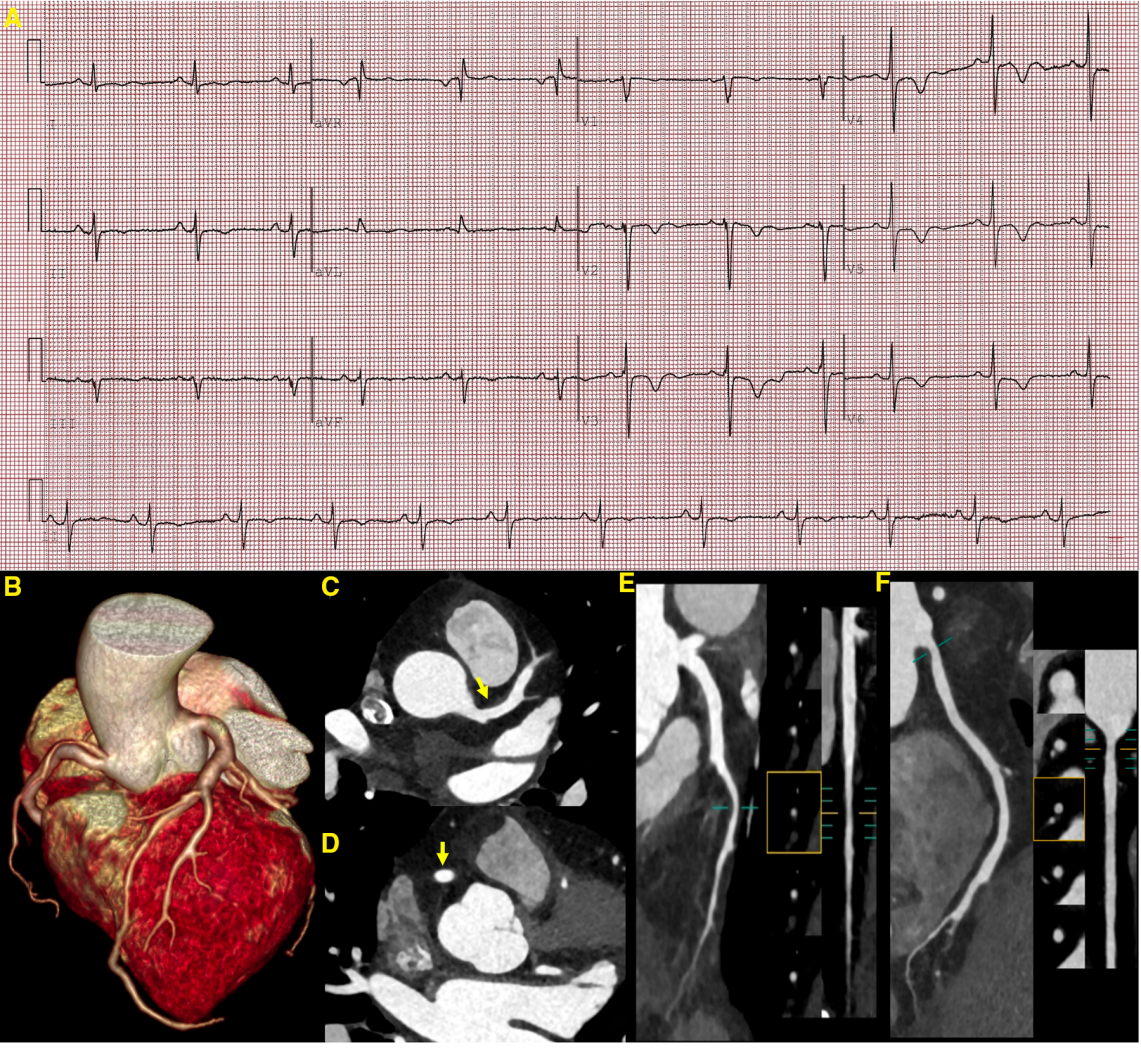
ECG and CCTA images demonstrating a sinus rhythm, a left deviation of the electrical axis, inverted T waves, and poor R-wave progression (**A**). A three-dimensional volume-rendered image (**B**) shows normal coronary artery alignment with continuous lumen. Axial images (**C**,**D**) show a mild diffuse dilatation of the LAD artery and RCA, with maximum diameters of 4.5 mm in the LAD artery and 4.7 mm in the RCA (yellow arrow). A curved planar reformation (**E**,**F**) demonstrates a non-calcified plaque in the proximal segment of the LAD artery and RCA with mild luminal narrowing. LAD, left anterior descending; RCA, right coronary artery.

**Figure 4:**
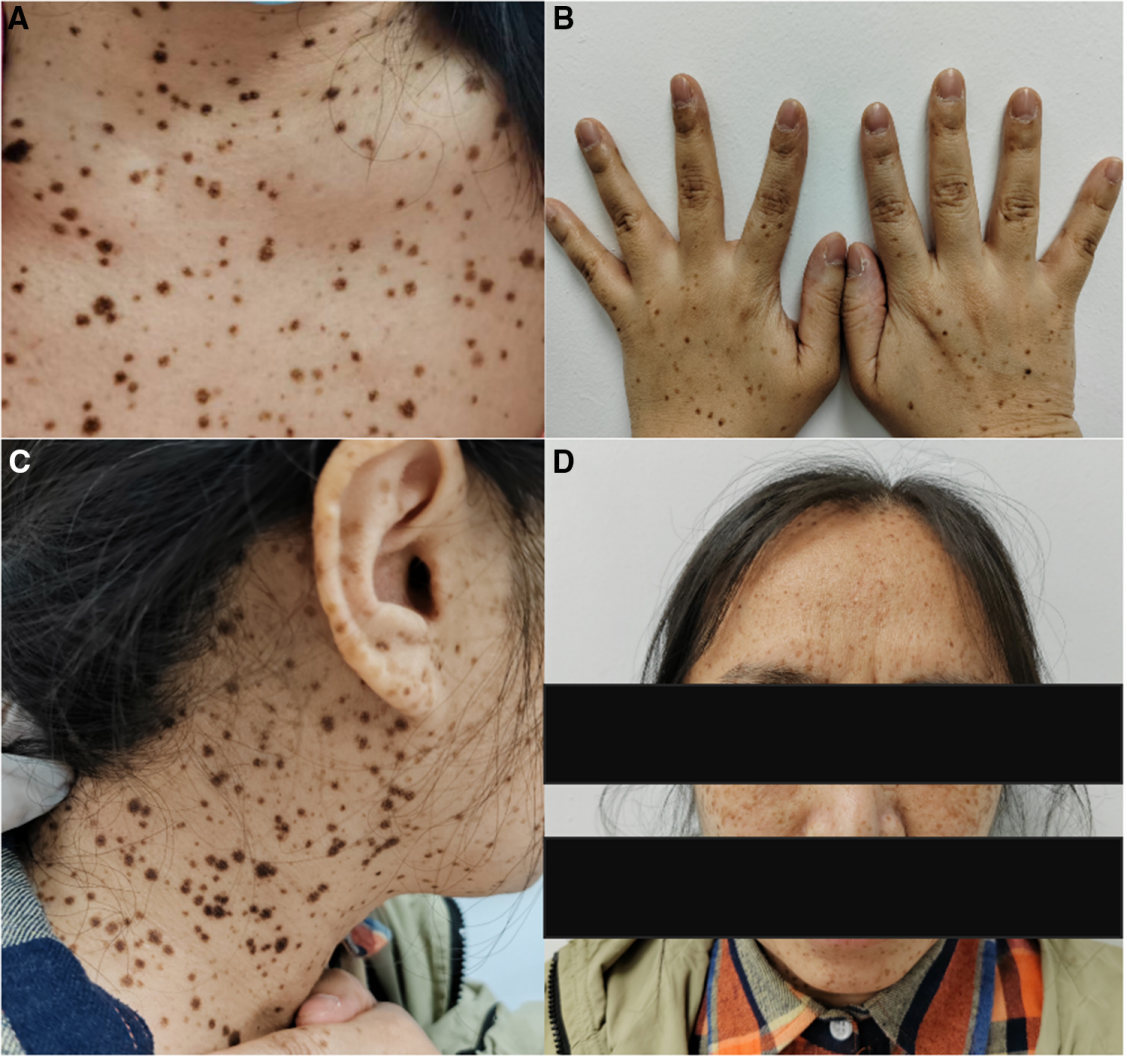
The patient presents multiple lentigines dispersed mainly on the face (**D**), neck (**A**,**C**), and limbs (**B**), mostly flat with the skin, which gradually increase, reaching a peak in adolescence.

From the previous history of the patient, it became evident that she presented with abnormal electrocardiographic findings at the age of 2 years. A recent electrocardiogram (ECG) revealed a left-sided electrical axis deviation with inverted T waves (Figure 3). Since the age of 4, she has been presenting with multiple lentigines (brown macules) dispersed mainly on the face, neck, and limbs, mostly flat within the skin, which gradually increase over time and reach a peak in terms of both number and extent in adolescence (Figure 4). The cutaneous manifestation intensified and deepened during the summer months because of sunlight exposure, which led to the finding of intradermal nevus by the pathological examination. In recent years, she has been experiencing exertional dyspnea. The physical examination was unremarkable, as borne out by normal eye spacing (3.0 cm), cognitive and auditory functions, and no malformations of the skeletal and reproductive systems. An ultrasonographic evaluation of the genitourinary system indicated hydronephrosis in the left kidney and a Nabothian cyst in the cervix, with no additional abnormalities observed. A chest computed tomography showed no thoracic skeletal deformities or hydrothorax. The patient has no children. A screening showed no multiple lentigines or related symptoms in the immediate family, which excluded the clinical possibility of LS according to the study of Voron et al (5). Complete blood cell count, urine analysis, and serum carcinoembryonic antigen showed no abnormalities. Serum biochemical indices showed a slightly higher level of low-density lipoprotein cholesterol with a value of 3.71 mmol/L (0–3.64 mmol/L). The HCM-AF score was 14 (less than 17), meaning that the risk of the patient developing new-onset atrial fibrillation was low (6).

Given the patient’s clinical features and history, ECG, TTE, and CMR, a diagnosis of Leopard syndrome was highly likely (5). A supplemental genetic testing revealed a heterozygous missense mutation in the *PTPN11* gene (c.836A>G, p.Tyr279Cys), confirming its suspicion. Medical therapy (metoprolol or verapamil) was recommended to minimize cardiac burden and alleviate exertional dyspnea. For the multiple lentigines, intense pulsed light therapy was advised (7). However, the patient insisted that her current clinical presentation was not severe enough to affect her daily life and therefore refused further treatment with full awareness of the adverse cardiovascular events that may occur. We followed up the patient closely, and after 3 months, she developed transient chest pain that resolved spontaneously. Cardiac biomarkers such as troponin T (0.047 ng/ml), troponin I (0.051 g/L), creatine kinase isoenzyme (12 U/L), and natriuretic peptide (12 pg/ml) did not show any significant abnormality in the first 24 h after admission. A supplemental CCTA revealed minor non-calcified plaques in the left anterior descending artery and right coronary artery with mild luminal stenosis, which was unlikely to cause episodes of chest pain. In addition, a mild diffuse dilatation of the left anterior descending and right coronary arteries was noted (Figure 3). However, apical hypertrophy was unchanged from the CMR imaging study performed three months earlier (Figure 2). Unfortunately, the patient again refused further treatment (Table 1).

**Table 1:** Timeline.

Sequence of events	First admission	Three-month follow-up
Presentation	Exertional dyspnea	■ Exertional dyspnea ■ Transient chest pain that resolved spontaneously
Multimodality imaging	■ ECG revealed a left-sided electrical axis deviation with inverted T waves ■ TTE detected a suspected apical tumor ■ CMR identified it as focal left ventricular hypertrophy	■ CCTA revealed minor non-calcified plaques with mild luminal stenosis and a mild diffuse dilation of the bilateral coronary arteries ■ CMR showed no change
Physical examination	Multiple lentigines	Multiple lentigines
Laboratory tests	No abnormalities (routine blood, urine analysis, and serum carcinoembryonic antigen)	No abnormalities (cardiac biomarkers)
Other screening tests	No abnormalities	No abnormalities
Treatment	Medical therapy and intense pulsed light (refused)	Medical therapy and intense pulsed light (refused)

ECG = electrocardiogram; TTE = transthoracic echocardiogram; CMR = cardiac magnetic resonance; CCTA = Coronary computer tomography angiography.

## Discussion

LS is caused by a missense mutation in the *PTPN11* gene encoding the protein tyrosine transferase Shp2, and its most common cardiac manifestation, LVH, may be attributed to mutations in exons 7 and 12 on the *PTPN11* gene (8). It has been found that LVH often precedes the formation of multiple freckles (1, 9). LVH caused by LS is more likely to result in arrhythmias or outflow tract obstruction, both strongly associated with adverse cardiovascular events (1, 10). Previous research has documented instances of sudden cardiac death or cardiac arrest related to LVH in patients with LS (11–13). However, for LS patients with only mild cardiac abnormalities, long-term prognosis appears to be favorable (1). This is similar to familial hypertrophic cardiomyopathy (14). Other cardiac abnormalities in LS, such as LV outflow tract obstruction and non-sustained ventricular tachycardia, are also associated with adverse cardiac events (13). Therefore, early intervention in the disease may significantly impact its prognosis. In addition, pulmonary stenosis is the most common cardiac structural abnormality in patients with LS, with a prevalence rate ranging from 10% to 40%, while ECG abnormalities indicating underlying cardiac structural anomalies occur in approximately 75% of patients with LS (1, 5, 15, 16). Diffuse coronary artery dilatation is rare and has been seen in only a few reported cases (17, 18).

In our patient, TTE showed a predominantly hypoechoic LV apical mass. The echogenicity of the lesion was unusual for normal myocardium, and its form resembled a well-defined protrusion toward the pericardium rather than the “spade-like” formation, which is the classic hallmark of apical hypertrophy (19, 20), giving the impression of an apical mass-like lesion. For further characterization of the apical mass-like lesion, CMR imaging was recommended. As there was no prior history of malignancies in the patient, metastases were ruled out. Primary cardiac tumors have a diverse composition and often show signal inhomogeneity, usually revealing an iso-to-low signal at T1-weighted CMR, an iso-to-high signal at T2-weighted imaging, and a marked delayed enhancement at LGE compared with a normal myocardium (21, 22). All CMR sequences in this patient showed an abnormal apical morphology with a lesion length of approximately 17 mm at the end of diastole and myocardial tissue characteristics consistent with normal myocardial tissue and a normal contraction pattern. A maximum end-diastolic myocardial thickness of ≥15 mm and inverted T waves serve as diagnostic criteria for LVH (23). Recent research demonstrated that hypertrophy occurring in the apical region with a thickness of ≥11 mm was diagnostic (24). Although echocardiography is traditionally the imaging modality of choice for LVH, CMR may offer better imaging options for the diagnosis and characterization of focal apical hypertrophy because of its high spatial resolution and complete tomographic coverage of the ventricles, in particular the apical region of the LV, which may be difficult to evaluate by TTE (25, 26). In addition, CMR allows a multiparametric imaging of the myocardium to fully assess tissue characteristics, which may be helpful in the differential diagnosis of various mass-like lesions (27, 28). The limitations of CMR, however, are the long examination time and the frequent breath-holds. Recently, all-in-one and real-time CMR has been envisioned as a means of addressing these issues, allowing CMR to hopefully lead to efficient imaging in the future (29, 30).

The patient's clinical symptoms were atypical, presenting only as shortness of breath after activity, leading to her undiagnosed condition. During the follow-up period, she experienced transient apical pain that relieved spontaneously, and CTA did not show severe coronary artery stenosis. Despite no progression in the lesion observed in a 3-month follow-up CMR, early intervention to prevent irreversible cardiac damage remains paramount. Rapamycin has shown promise in reversing PTPN11 mutant hypertrophic cardiomyopathy in mouse models by inhibiting the mTOR pathway; however, its application in human subjects requires further clinical validation (31, 32). Symptomatic treatment continues to be the most common treatment modality in clinical practice, with beta-blockers or non-dihydropyridine calcium channel blockers recommended for managing symptomatic non-obstructive myocardial hypertrophy to reduce chest pain and enhance exercise capacity. In the absence of any improvement with medication, surgical removal of the left ventricular outflow obstruction is indicated for obstructive myocardial hypertrophy (15, 16, 33, 34). Although the patient declined further treatment, we will maintain rigorous monitoring and strive to secure her consent for prompt intervention.

This case report shows the value of multimodality imaging by echocardiography, cardiac magnetic resonance imaging, and coronary computed tomography to unravel the diagnostic challenges posed by unusual cardiac manifestations (apical hypertrophy, coronary dilatation) in complex disorders such as Leopard syndrome.

## Data Availability

The original contributions presented in the study are included in the article/**Supplementary Material**, further inquiries can be directed to the corresponding author.
